# Clinical validation of T1ρ mapping for the assessment of hepatic fibrosis in patients with chronic liver disease

**DOI:** 10.1007/s00330-025-12225-5

**Published:** 2025-12-20

**Authors:** Narine Mesropyan, Johannes Chang, Philipp Lutz, Felix Kaiser, Oliver M. Weber, Christoph Katemann, Tatjana Dell, Dmitrij Kravchenko, Can Yueksel, Daniel Kuetting, Claus C. Pieper, Julian A. Luetkens, Alexander Isaak

**Affiliations:** 1 Department of Diagnostic and Interventional Radiology, University Hospital Bonn, Bonn, Germany; 2 Department of Internal Medicine I and Cirrhosis Center Bonn, University Hospital Bonn, Bonn, Germany; 3 Philips GmbH, Hamburg, Germany; 4 Department of Diagnostic and Interventional Radiology, University Hospital Aachen, Aachen, Germany

**Keywords:** Magnetic resonance imaging, Quantitative mapping, Hepatic fibrosis

## Abstract

**Objectives:**

To evaluate the diagnostic utility of T1ρ mapping for assessing hepatic fibrosis in patients with chronic liver disease (CLD), including steatotic liver disease (SLD).

**Materials and methods:**

In this prospective study (September 2024 to May 2025), consecutive patients with CLD underwent liver MRI, including MR-elastography, PDFF, T1, extracellular volume fraction (ECV), and T1ρ mapping. MRE-based liver stiffness was used as the reference to assess the diagnostic performance of the MRI-derived mapping parameters. MR-elastography-based liver stiffness thresholds for significant fibrosis (≥ F2) were set at > 3.66 kPa for participants without hepatic steatosis (PDFF ≤ 5%), and > 3.14 kPa for participants with hepatic steatosis (PDFF > 5%). The *t*-test, Spearman’s correlation, and the ROC analysis were applied.

**Results:**

One hundred twelve CLD participants were included (mean age, 48 ± 16 years*;* 53 participants with hepatic steatosis). All assessed quantitative mapping parameters were significantly increased in participants with significant fibrosis than in those without (e.g., T1ρ: 110 ± 15 vs 92 ± 6 ms, *p* < 0.001). T1ρ revealed a moderate to strong correlation with MR-elastography-based stiffness, superior to T1 and ECV (entire cohort: *r* = 0.75 [T1ρ] vs 0.49 [native T1] vs 0.68 [ECV]; participants with hepatic steatosis: *r* = 0.67 [T1ρ] vs 0.32 [native T1] vs 0.62 [ECV]; *p* < 0.05 in each case, respectively). T1ρ provided the highest diagnostic performance for diagnosing significant fibrosis (in the entire cohort: AUC 0.90 [T1ρ] vs 0.73, *p* < 0.001 [native T1], vs 0.81, *p* = 0.05 [ECV]; in participants with hepatic steatosis: AUC 0.87 [T1ρ] vs 0.67, *p* = 0.03 [native T1], vs 0.79, *p* = 0.047 [ECV], *p* values are given vs T1ρ).

**Conclusion:**

Hepatic T1ρ might be a more accurate marker of hepatic fibrosis in CLD, including SLD, compared to hepatic native T1 and ECV mapping.

**Key Points:**

***Question*** *Accurate non-invasive assessment of hepatic fibrosis remains challenging, particularly in the presence of steatosis, where MRI biomarkers such as native T1 and ECV fraction are limited*.

***Findings*** *T1ρ mapping outperformed native T1 and ECV for identifying significant fibrosis and maintained robust accuracy in the presence of hepatic steatosis*.

***Clinical relevance*** *T1ρ mapping offers a robust, non-invasive MRI biomarker for assessing hepatic fibrosis across the full spectrum of CLD, including SLD, with superior accuracy to native T1 and ECV and reduced influence from hepatic fat infiltration*.

**Graphical Abstract:**

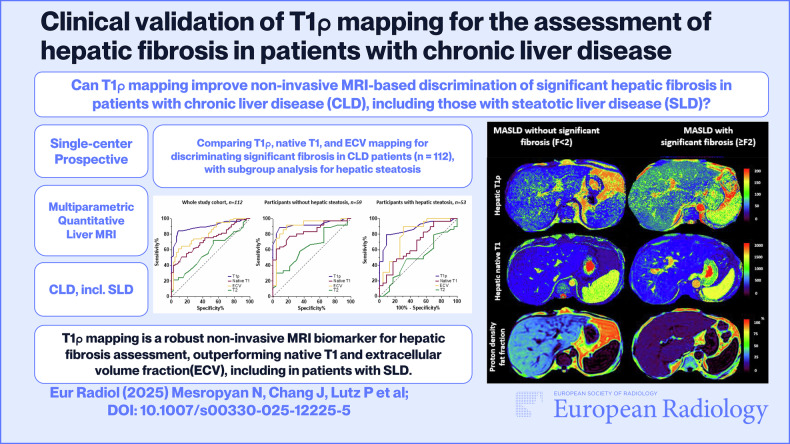

## Introduction

Hepatic fibrosis is the most critical prognostic determinant of liver-related morbidity and mortality, with risk escalating progressively with each fibrosis stage. This is particularly true in the presence of ongoing inflammation and hepatocellular injury, as observed, for example, in at-risk MASH [1]. Thus, early diagnosis and follow-up of hepatic fibrosis in chronic liver disease (CLD) patients, including those with steatotic liver disease (SLD), is pivotal.

To address this need, various non-invasive methods have been developed and proposed, in addition to liver biopsy. These approaches encompass serological markers and panels, as well as imaging (ultrasound, MRI, and CT). Serological biomarkers are easy to obtain and cost-effective; however, their specificity may be limited due to the influence of various factors, including the etiology of CLD and extrahepatic conditions (e.g., renal failure, medication) [2]. In CLD management, imaging plays a key role, particularly in ruling out malignancy and assessing associated complications. MR-elastography (MRE) is currently considered the most accurate, non-invasive alternative to liver biopsy for liver fibrosis assessment [3, 4]. MRE quantitatively measures liver stiffness and demonstrates strong concordance with histologic fibrosis staging, achieving pooled area under the receiver operating characteristic (ROC) curve values of 0.88–0.93 for ≥ F2 fibrosis and up to 0.94 for cirrhosis according to large-scale meta-analyses [3, 4]. Yet, it requires additional expensive hardware and patient preparation. Quantitative MRI, including T1 and ECV mapping, is an emerging approach that holds promise for the non-invasive assessment of hepatic fibrosis and disease severity [5–9]. However, its diagnostic accuracy is limited in the presence of hepatic steatosis, which is important due to the frequency of SLD [1, 10–12]. To overcome this limitation, various technical adaptations of T1 mapping have been attempted, though they remain technically demanding and lack thorough validation [12–14]. Another recently proposed, potentially more accurate, and robust alternative to conventional T1 and ECV mapping is T1ρ mapping. A previous preclinical study has shown that T1ρ is an accurate biomarker of hepatic fibrosis and portal hypertension in various models of SLD, outperforming conventional T1, ECV, and T2 mapping [15].

Building on these advancements, our study aimed to clinically translate these findings and assess the diagnostic utility of T1ρ mapping for assessing hepatic fibrosis across the entire CLD spectrum, including SLD. Diagnostic performance of T1ρ was compared with that of T1, ECV, and T2 mapping, using MRE-based liver stiffness as the standard of reference.

## Materials and methods

The study received ethical approval (no. 155/23-EP), and all participants provided written informed consent prior to MRI. From September 2024 to May 2025, consecutive patients with CLD referred for liver MRI based on clinical indications were enrolled. Routine laboratory blood tests were performed using standardized procedures. Following non-invasive scores for liver fibrosis and disease severity assessment were subsequently calculated: the fibrosis-4 index, the aspartate aminotransferase-to-platelet ratio index, the aspartate aminotransferase to alanine aminotransferase ratio (de Ritis), and the Model for End-Stage Liver Disease. All clinical and laboratory data were extracted from the patients’ medical records. In addition, hematocrit values required for ECV calculation were obtained prior to MRI examination.

### Liver MRI

MRI examinations were carried out on a clinical 1.5-Tesla whole-body scanner (Ingenia, Philips Healthcare, The Netherlands), utilizing a 28-channel body coil with digital signal reception capability. Alongside standard clinical sequences, MRE, proton density fat fraction (PDFF), T1, T2, and T1ρ mapping were acquired.

For PDFF quantification, a six-echo spoiled gradient-echo Dixon sequence (mDixon Quant) was employed, as described elsewhere [12]. A 2D gradient-recalled echo sequence with integrated cyclic motion-encoding gradients was utilized for MRE, as described elsewhere [7, 8, 12]. For hepatic T2 mapping, a six-echo gradient spin echo sequence (GraSE) was acquired as described elsewhere [7, 8, 12]. For T1 mapping, a standard modified Look-Locker inversion recovery (MOLLI) sequence before and after contrast media injection was applied (TR: 3.8 ms; TE: 1.85 ms; flip angle 10°; acquired voxel size: 1.98 × 2.46 × 10 mm; reconstructed voxel size: 1.13 × 1.13 × 10 mm) [7, 8]. T1ρ mapping was performed using a single-shot balanced steady-state free precession turbo field echo sequence (repetition time: 4.1 ms; echo time: 1.88 ms; flip angle: 20°) incorporating an adiabatic T1ρ preparation module with spin-lock times of 0, 30, and 60 ms and a spin-lock pulse frequency of 500 Hz to achieve T1ρ weighting. To ensure full magnetization recovery, a pause of 4 s was applied between each spin-lock acquisition. The acquired spatial resolution was 2 × 2 × 1 mm³, with reconstructed resolution of 1.17 × 1.17 × 10 mm³, and a field of view of 300 × 300 mm². All quantitative maps were acquired in a single matched slice positioned at the level of the portal vein bifurcation, while the participants were instructed to hold their breath.

### Imaging analysis

Image analysis was conducted using commercially available software (Picture Archiving and Communication System software, DeepUnity Diagnost, Agfa; Advanced Visualization Workspace, Version 11, Philips Healthcare). Image analysis was performed by a board-certified radiologist (NM) with six years of experience in abdominal imaging and quantitative mapping, in random order and blinded to clinical and laboratory data. Hepatic T1, T1ρ, T2 relaxation times, and PDFF were analyzed using quantitative maps automatically reconstructed at the MRI console. For the quantitative assessment of relaxation times and PDFF, the mean value from manually drawn circular or oval regions of interest (ROIs) was calculated. Each ROI measured at least 1 cm² and was positioned within the liver parenchyma—typically in the left, right, and central lobes (segment IVa or IVb)—while carefully avoiding image artifacts, vessels, and bile ducts. In most cases, three representative ROIs were placed to ensure adequate and representative sampling of the liver parenchyma across different segments. The mean value of all ROIs was used for subsequent analysis. ECV was corrected for hematocrit and calculated from pre-contrast and post-contrast T1-relaxation times (acquired 10 min after contrast administration) according to the following formula: (1 − hematocrit) × (1/T1_liver parenchyma post-contrast_ − 1/T1_liver parenchyma pre-contrast_)/(1/T1_aortic post-contrast_ − 1/T1_aortic pre-contrast_) [5–8]. MRE-based liver tissue stiffness was obtained from the stiffness confidence map by placing the largest possible ROI covering the right and left lobes, also avoiding organ borders and image artifacts. For inter- and intra-observer reproducibility assessment, 50 MRI examinations were randomly selected from the study cohort to provide a representative subset for reliability analysis while ensuring methodological feasibility.

For the assessment of diagnostic performance of T1, ECV, T1ρ, and T2 mapping parameters for the detection of significant fibrosis, the entire study cohort was dichotomized into participants without significant fibrosis (F < 2) and with significant fibrosis (F ≥ 2) based on MRE-derived liver stiffness. Significant fibrosis was defined by a cutoff of 3.66 kPa in CLD patients without hepatic steatosis (defined as PDFF ≤ 5%) and 3.14 kPa in those with hepatic steatosis (defined as PDFF > 5%) [3, 4]. Different cutoff values were selected to allow for more accurate fibrosis assessment based on previously published studies [3, 4].

### Statistical analysis

Commercially available statistical software packages were used to perform the analyses (Prism, Version 9, GraphPad Software; SPSS, Version 7, IBM SPSS Software; MedCalc, Version 23.1.5, MedCalc Software). The level of statistical significance was set at < 0.05. The Kolmogorov–Smirnov test was used to check for normal distribution. Descriptive statistics are provided as means with standard deviations or as absolute counts, as appropriate to the data type. Two-group comparisons were conducted using Student’s *t*-test or Mann–Whitney *U*-test, depending on data distribution. Non-parametric correlations were assessed using Spearman’s rank coefficient. Inter- and intra-reader agreement for each MRI-derived mapping parameter was assessed with interclass correlation coefficients (ICCs, < 0.5 = poor; 0.5 to < 0.75 = moderate; 0.75 to < 0.9 = good; ≥ 0.9 = excellent). Intra-reader agreement was evaluated only for one reader. The diagnostic performance of all quantitative mapping parameters for identifying significant fibrosis was assessed by generating ROC curves and calculating the corresponding areas under the curve (AUCs) using MRE-derived liver stiffness as the reference standard. The optimal threshold for each parameter was determined based on Youden’s index, which identifies the point on the ROC curve that maximizes the sum of sensitivity and specificity. The DeLong method was applied for pairwise comparison of AUCs [16].

## Results

**Fig. 1:**
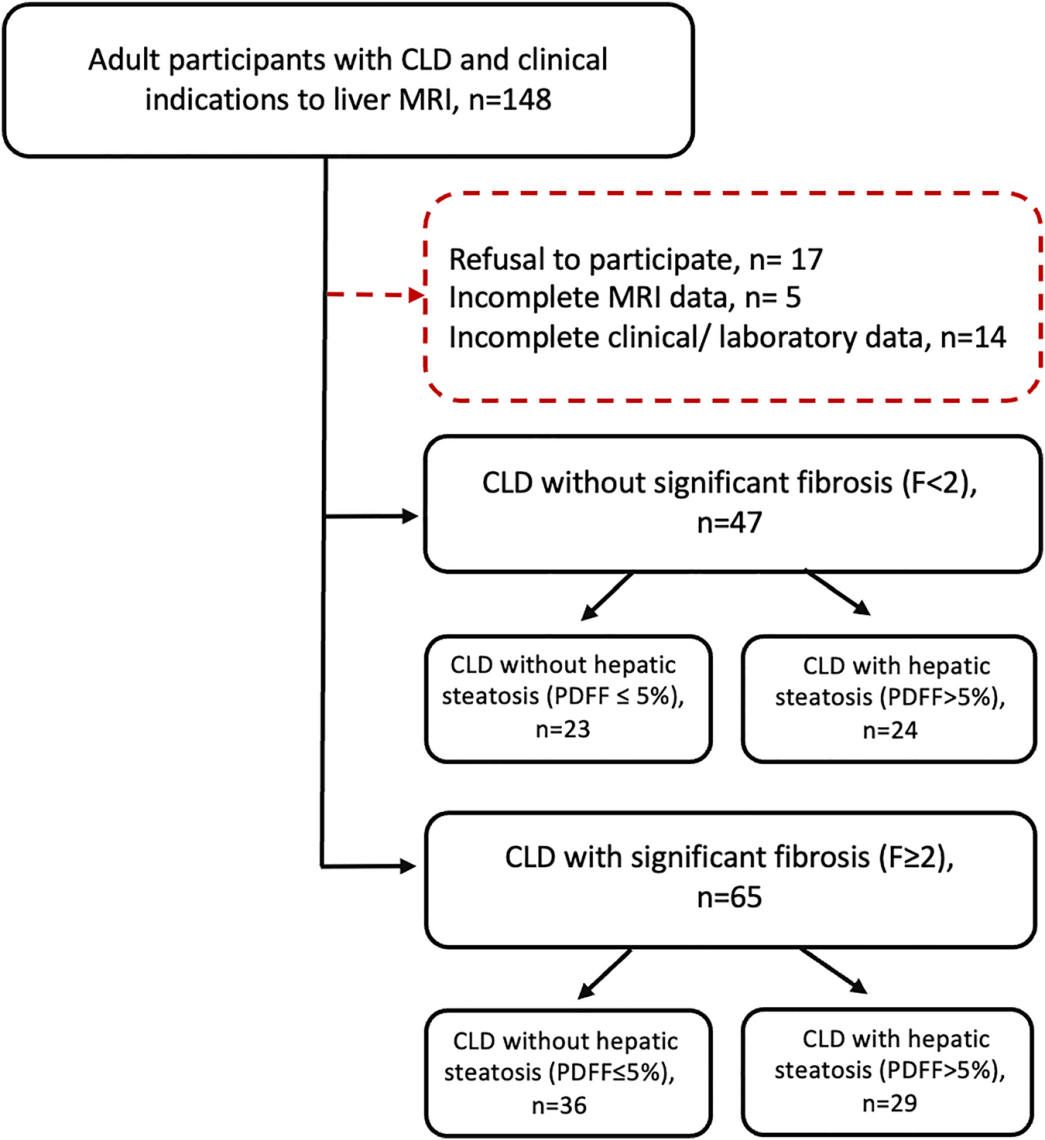
Flow diagram of participants’ inclusion and exclusion. CLD, chronic liver disease; PDFF, proton density fat fraction; F, fibrosis stage

After excluding patients who declined to participate, and those with insufficient clinical and/or MRI data, a total of 112 participants (mean age: 48 ± 16 years; range: 19–82 years; 46 female) were included in the final analysis. In the entire study cohort, 47/112 (42%) patients had no significant fibrosis (F < 2), and the remaining participants (65/112, 58%) had significant fibrosis (F ≥ 2). A total of 59/112 (53%) had no relevant hepatic steatosis(PDFF ≤ *5%*), and the remaining participants (53/112, 47%) had hepatic steatosis(PDFF > 5%). Detailed demographic, clinical, laboratory, and MRI characteristics of the included participants are provided in Table 1. The flow diagram of included patients is shown in Fig. 1.

**Table 1:** General, clinical, laboratory, and MRI characteristics of the participants with CLDs

Variable	Participants without significant fibrosis (F < 2, *n* = 47)	Participants with significant fibrosis (F ≥ 2, *n* = 65)	*p* value
General, clinical, and laboratory parameters			
Age (years)	48 ± 16	52 ± 14	0.17
Body mass index (kg/m^2^)	27 ± 5	28 ± 5	0.80
Sex			0.33
Male	25 (53%)	41 (63%)	
Female	22 (47%)	24 (37%)	
Etiology of CLD			
Autoimmune liver disease	11 (23%)	16 (25%)	0.99
Alcoholic LD	-	10 (15%)	0.004
MASLD	16 (34%)	11 (17%)	0.04
Viral hepatitis	2 (4%)	4 (6%)	0.99
Toxic	1 (2%)	4 (6%)	0.39
Unknown	10 (21%)	4 (6%)	0.02
Vascular	5 (11%)	9 (14%)	0.77
Others	2 (4%)	7 (11%)	0.29
Hematocrit level (%)	42 ± 4	41 ± 6	0.19
ALT (U/L)	46 ± 40	47 ± 31	0.88
AST (U/L)	37 ± 21	53 ± 42	0.02
GGT (U/L)	83 ± 92	149 ± 175	0.02
AP (U/L)	99 ± 54	140 ± 115	0.03
Bilirubin (mg/dL)	0.71 ± 0.51	1.33 ± 1.54	0.01
Albumin (g/L)	44 ± 5	42 ± 7	0.19
Platelets cells × 10^9^/L	237 ± 79	180 ± 109	0.003
C-reactive protein (mg/L)	3.9 ± 6.6	5.7 + 9.1	0.26
Creatinine (mg/dL)	0.83 ± 0.15	0.94 ± 0.56	0.24
International normalized ratio	1.02 ± 0.24	1.12 ± 0.27	0.07
GFR (mL/min/1.73 m^2^)	80 ± 14	77 ± 15	0.27
MELD	6 ± 1	9 ± 3	< 0.001
ASL/ALT (de-Ritis)	1.15 ± 0.48	1.04 ± 0.58	0.30
APRI	0.36 ± 0.25	0.87 ± 1.03	0.001
FIB-4	1.53 ± 1.27	3.41 ± 3.63	0.001
MRI-derived quantitative parameters			
MRE-derived liver stiffness (kPa)	2.7 ± 0.5	6.3 ± 2.1	< 0.001
PDFF (%)	11 ± 10	7 ± 7	0.03
T1ρ (ms)	92 ± 6	110 ± 15	< 0.001
Native T1 (ms)	530 ± 71	628 ± 129	< 0.001
ECV (%)	27 ± 7	37 ± 10	< 0.001
T2 (ms)	48 ± 5	50 ± 7	0.047

*F* fibrosis stage, *ALD* alcohol assotiated liver disease, *MELD* score model of end-stage liver disease, *ALT* alanine aminotransferase, *AST* aspartate aminotransferase, *AP* alkaline phosphatase, *GGT* gamma-glutamyltransferase, *APRI* aspartate aminotransferase to platelet ratio index, *FIB-4* fibrosis-4-score, *ASL/ALT (de-Ritis)* De-Ritis ratio

### Quantitative multiparametric liver MRI

**Fig. 2:**
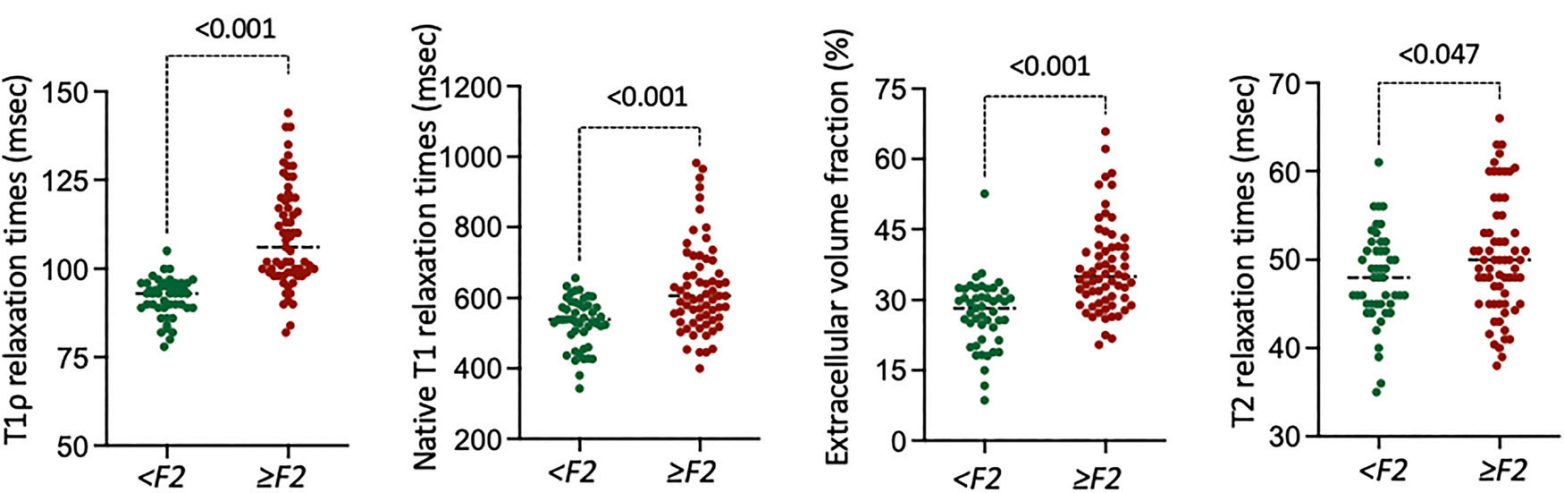
Individual values of hepatic T1ρ, native T1, ECV, and T2 are shown in dot plots, grouped according to the presence or absence of significant fibrosis (defined as F ≥ 2). Horizontal lines indicate the group means. F, fibrosis stage

**Fig. 3:**
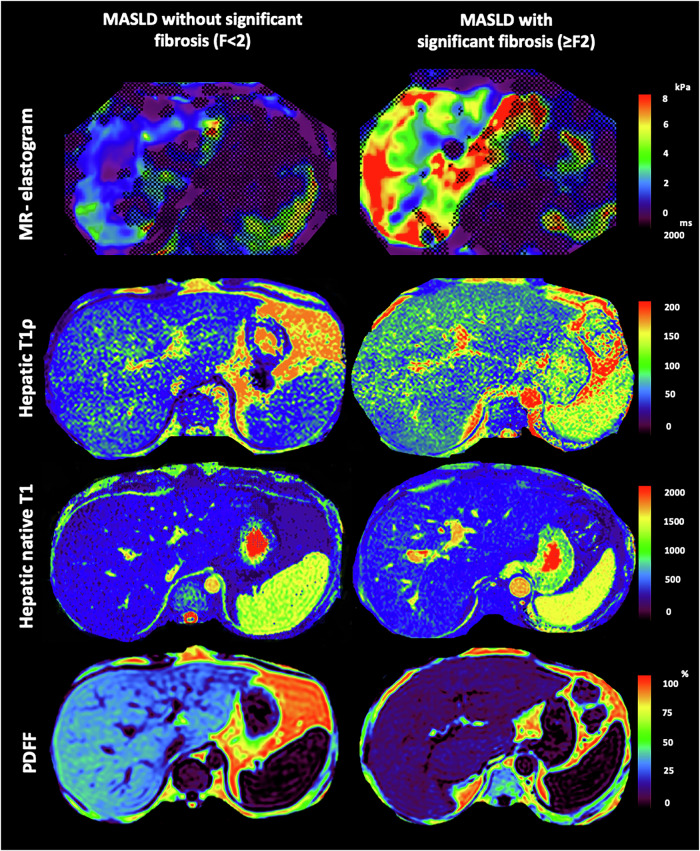
MRI images illustrating cases of metabolic dysfunction-associated steatotic liver disease patients (MASLD) with and without significant fibrosis (defined as fibrosis stage F ≥ 2 and F < 2, respectively). Due to opposing effects of hepatic steatosis and fibrosis on native T1 relaxation times, T1 values may appear pseudonormalized, potentially masking the presence of significant fibrosis in affected individuals. In contrast, hepatic T1ρ appears more robust in the context of steatosis, allowing more reliable assessment of significant fibrosis. F, fibrosis stage

**Fig. 4:**
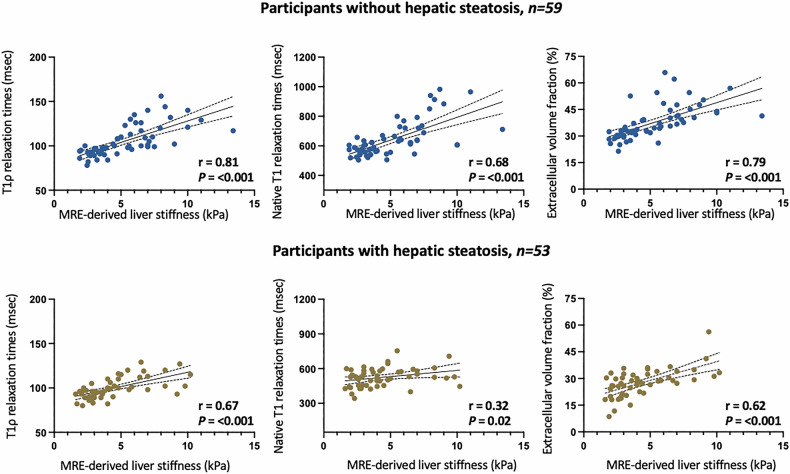
Scatter plots show correlations between MRE-based liver stiffness measurements and hepatic T1ρ, hepatic native T1, extracellular volume fraction. Regression lines are shown with corresponding 95% confidence intervals

Participants with significant liver fibrosis (F ≥ 2) showed an elevation in all hepatic mapping parameters under investigation compared to those without significant fibrosis (F < 2), e.g., T1ρ: 110 ± 15 ms vs 92 ± 6 ms, *p* < 001 (Table 1 and Figs. 2 and 3). Hepatic T1ρ mapping demonstrated moderate to strong correlations with MRE based liver stiffness measurements (entire study cohort: *r* = 0.75 (*p* < 0.001); patients with no hepatic steatosis: *r* = 0.81 (*p* < 0.001); patients with hepatic steatosis: *r* = 0.67 (*p* < 0.001)). The correlation coefficients were superior to those of hepatic native T1 and ECV (Table 2 and Fig. 4). In the subgroup analyses, hepatic native T1, ECV, and T1ρ showed all lower correlations in patients with hepatic steatosis compared to those without hepatic steatosis, with the most pronounced reduction observed for hepatic native T1 (*r* = 0.67 vs 0.32), followed by ECV (*r* = 0.79 vs 0.67) and T1ρ (*r* = 0.80 vs 0.67).

**Table 2:** Correlation matrix between MRI-derived quantitative mapping parameters and MRE-derived liver stiffness in participants with CLD, calculated in the whole study cohort (*n* = 112) and separately in CLD participants without hepatic steatosis (*n* = 59) and in CLD participants with hepatic steatosis (*n* = 53)

Variable	All patients with CLD (*n* = 112)	CLD without hepatic steatosis (PDFF ≤ *5%*, *n* = 59)	CLD with hepatic steatosis (PDFF > 5%, *n* = 53)
T1ρ	0.75 (< 0.001)	0.81 (< 0.001)	0.67 (< 0.001)
Native T1	0.49 (< 0.001)	0.68 (< 0.001)	0.32 (0.02)
ECV	0.68 (< 0.001)	0.79 (< 0.001)	0.62 (< 0.001)
T2	0.23 (0.001)	0.34 (0.01)	0.03 (0.82)

*p* values are given in parentheses

*CLD* chronic liver disease, *PDFF* proton density fat fraction, *ECV* extracellular volume fraction

Inter-reader agreement by the assessment of all quantitative mapping parameters was good and ranged from 0.767 to 0.838 (*p* < 0.001, in each case). Intra-reader agreement was good, with ICC values ranging from 0.821 to 0.896 (*p* < 0.001 for all) (Appendix S1).

**Fig. 5:**
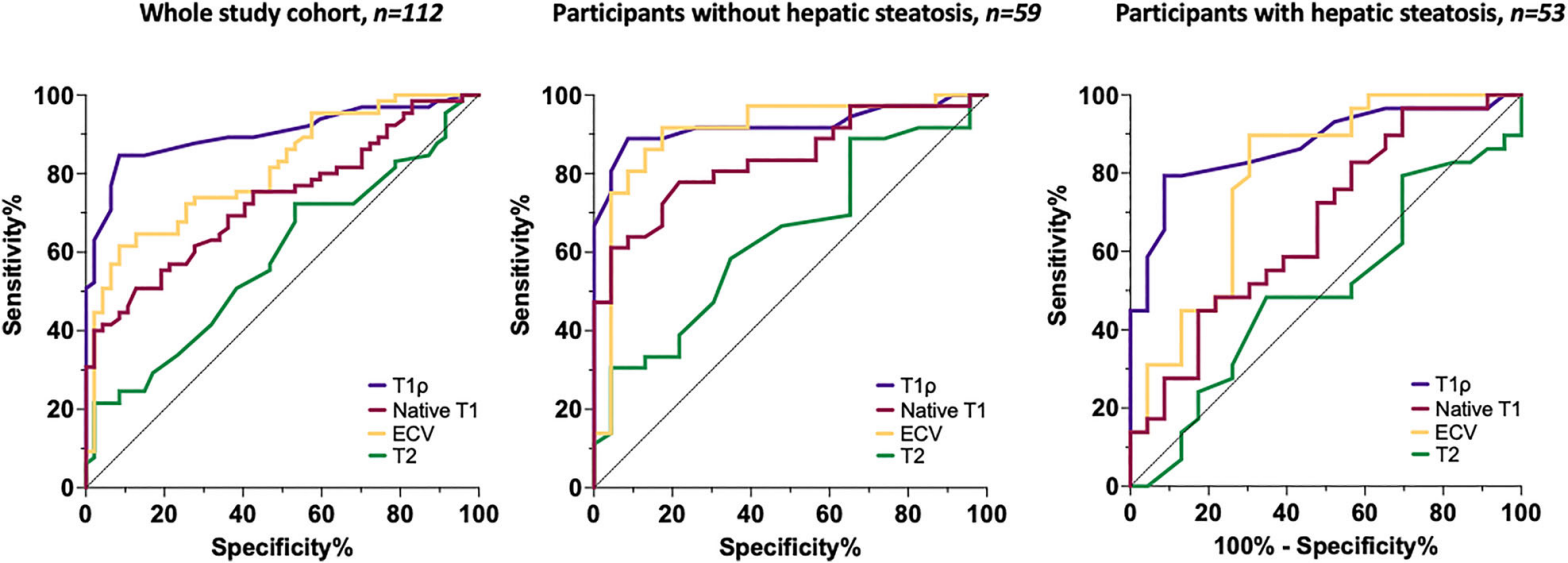
ROC analysis illustrating the diagnostic performance of quantitative MRI mapping parameters for detecting significant fibrosis (defined as fibrosis stage F ≥ 2). Separate curves are shown for hepatic T1ρ, native T1, ECV fraction, and T2 relaxation times

Within the entire study cohort, hepatic T1ρ revealed the highest diagnostic performance in discriminating significant fibrosis (AUC: 0.90, confidence interval [CI]: 0.84; 0.96, sensitivity of 84.6% [CI: 73.9; 91.4], specificity of 91.5% [CI: 80.1; 96.6] which was superior to that of hepatic native T1 (AUC: 0.73 [CI: 0.64; 0.82], *p* = 0.001) and ECV (AUC: 0.81 [CI: 0.73, 0.89] *p* = 0.05), *p* values are given vs T1ρ. In subgroup analysis, in patients with hepatic steatosis, this also remained true with AUCs as follows: 0.87 [T1ρ] vs 0.67, *p* = 0.03 [native T1] vs 0.79, *p* = 0.047 [ECV], *p* values are given vs T1ρ. In a cohort with no relevant hepatic steatosis, the diagnostic performance of T1ρ was similar to that of native T1 (*p* = 0.09) and ECV (*p* = 0.77), see also Table 3 and Fig. 5.

**Table 3:** Diagnostic performance of different quantitative MRI parameters for the detection of significant liver fibrosis (F ≥ 2) in participants with CLD

Variable	AUC	Cutoff value	Sensitivity (%)	Specificity (%)	NPV	PPV	Accuracy	*p* value
Whole study cohort (*n* = 112)								
T1ρ (ms)	0.90 (0.84, 0.96)	> 97.5	84.6 (73.9, 91.4)	91.5 (80.1, 96.6)	81.1 (68.6, 89.4)	93.2 (83.8, 97.3)	87.5 (80.1, 92.4)	< 0.001
Native T1 (ms)	0.73 (0.64, 0.82)	> 554	70.8 (58.8, 80.4)	59.6 (45.3, 72.4)	60.9 (46.5, 73.6)	71.2 (59.4, 80.7)	67.0 (57.8, 75.0)	< 0.001
ECV (%)	0.81 (0.73, 0.89)	> 30.4	73.9 (62.1, 82.9)	72.3 (58.2, 83.1)	66.0 (52.2, 77.6)	77.4 (63.4, 79.8)	72.3 (63.4, 79.8)	< 0.001
T2 (ms)	0.58	-	-	-	-	-	-	0.13
CLD without hepatic steatosis (PDFF ≤ 5%, *n* = 59)								
T1ρ (ms)	0.92	> 97.5	88.9 (74.7, 95.6)	91.3 (73.2, 98.5)	84.0 (65.3, 93.6)	94.1 (80.9, 98.4)	89.8 (79.5, 95.3)	< 0.001
Native T1 (ms)	0.84 (0.74, 0.94)	> 588.5	80.6 (64.9, 90.3)	69.6 (49.1, 84.4)	69.6 (49.1, 84.4)	80.6 (65.0, 90.2)	76.3 (64.0, 85.3)	< 0.001
ECV (%)	0.91 (0.82, 0.99)	> 34.1	80.6 (64.9, 90.3)	91.3 (73.2, 98.5)	75.0 (56.6, 87.3)	93.5 (79.3, 98.2)	84.7 (73.5, 91.8)	< 0.001
T2 (ms)	0.64	-	-	-	-	-	-	0.07
CLD with hepatic steatosis (PDFF > 5%, *n* = 53)								
T1ρ (ms)	0.87 (0.77, 0.97)	> 97.5	79.3 (61.6, 90.2)	91.3 (73.2, 98.5)	76.9 (57.9, 89.0)	85.2 (67.5, 94.1)	81.1 (68.6, 89.4)	< 0.001
Native T1 (ms)	0.67 (0.52, 0.818)	> 528.5	58.6 (40.7, 74.5)	60.9 (40.8, 77.8)	53.8 (35.5, 71.2)	63.0 (44.2, 78.5)	58.5 (45.1, 70.7)	0.04
ECV (%)	0.79 (0.67, 0.92)	> 27.3	75.9 (57.9, 87.8)	73.9 (53.5, 87.5)	70.4 (51.5, 84.1)	80.8 (62.1, 91.5)	75.5 (62.4, 85.1)	< 0.001
T2 (ms)	0.50		-	-	-	-	-	0.99

Data in parentheses are 95% confidence interval

*CLD* chronic liver disease, *AUC* area under the curve, *PPV* positive predictive value, *NPV* negative predictive value, *ECV* extracellular volume fraction

## Discussion

There is an unmet need for non-invasive, accurate tools for the assessment of hepatic fibrosis—the main driver of CLD progression. Hepatic steatosis, which is common in CLD patients, in particular those with SLD, is a relevant confounder for the mostly used quantitative mapping approaches, limiting their accuracy [17]. In a previous preclinical study assessing MRI-based quantification of fibrosis in different animal models of SLD, T1ρ was shown to be a more accurate and robust alternative to conventional T1, ECV, and T2 mapping. This prospective study aimed to clinically translate these findings in patients with the whole spectrum of CLD, including SLD, using MRE as the reference. The main finding of our study were: (1) T1ρ mapping showed moderate to strong correlations with MRE-based liver stiffness measurements, which were superior to that of hepatic native T1 and ECV., e.g., in cohort with hepatic steatosis (*r* = 0.67 [T1ρ] vs 0.32 [native T1] vs 0.62 [ECV]); (2) T1ρ mapping outperformed hepatic native T1 and ECV in discrimination significant fibrosis in cohort with hepatic steatosis with AUC of 0.87 (CI: 0.77, 0.96), sensitivity of 79.3% (CI: 61.6; 90.2) and specificity of 91.3% (73.2; 98.5).

T1ρ mapping reflects the interactions between motion-restricted water molecules and their local macromolecular environment. Due to its high sensitivity to low-frequency motional processes, it enables the assessment of macromolecular composition and proton exchange dynamics within biological tissues [18, 19]. Liver fibrosis is characterized by the excessive deposition of macromolecules, including collagen and other extracellular matrix components. Therefore, T1ρ mapping might be a potential biomarker for hepatic fibrosis assessment. In several prior preclinical studies, T1ρ relaxation times have been associated with liver fibrosis and hepatic injury in animal models of biliary duct ligation and carbon tetrachloride-induced liver injury [20–23]. These findings were corroborated in a recent histopathological validation study in rats in different animal models of SLD [15]. To date, only a limited number of clinical studies have attempted to translate these findings into the clinical setting, yielding inconsistent results. On the one hand, most prior human studies have primarily focused on cohorts with advanced liver disease and cirrhosis, in which hepatic steatosis was not expected [24, 25]. On the other hand, T1ρ may reflect hepatic function rather than serving as a fibrosis biomarker [26]. Thus, further validation of this technique in a larger cohort within the whole spectrum of CLD is necessary.

Clinically translating findings from a recent preclinical study in SLD models, we could confirm a moderate to strong correlation between T1ρ mapping and hepatic fibrosis assessed by MRE in CLD patients, including those with SLD. This correlation was also stronger than that observed with hepatic native T1 and ECV in the entire study cohort, in which 47% of participants had hepatic steatosis. Subgroup analysis of participants with hepatic steatosis further supported this observation. As expected, hepatic fat deposition led to shortened T1 relaxation times, resulting in inaccurate T1 measurements and, therefore, weaker correlation between T1 and ECV with MRE-based liver stiffness in patients with hepatic steatosis compared to those without. Translating these results clinically would mean potential overestimation or underestimation of disease severity in SLD using T1 and ECV mapping. In contrast, T1ρ mapping demonstrated robust moderate-to-strong correlations with MRE-derived liver stiffness both in the entire study cohort and in subgroups with and without hepatic steatosis. These findings are consistent with previously reported, histologically validated data in rats [15]. In our study cohort, ECV performed well, showing a moderate correlation with MRE-based liver stiffness in patients with hepatic steatosis. Although this correlation was lower than that of T1ρ mapping, it was higher than that of hepatic native T1. This may be attributed to the fact that ECV is a physiologically normalized measure and has additional dependencies and terms, which make it more robust compared to native T1.

Hepatic T1ρ mapping demonstrated the highest diagnostic performance in identifying significant fibrosis assessed by MRE, outperforming both hepatic native T1 and ECV. While the diagnostic performance of hepatic native T1 and ECV declined in the presence of hepatic steatosis, T1ρ mapping maintained consistently high diagnostic performance across all cohorts. These findings suggest that T1ρ mapping is likely less affected by hepatic fat deposition, in line with a recent histological validation study [15].

Despite significant differences in T2 mapping between patients with and without significant fibrosis, and a correlation with hepatic fibrosis in patients without steatosis, T2 mapping did not correlate with MRE-based liver stiffness in the cohort with hepatic steatosis only. T2 mapping was not able to discriminate significant fibrosis in our study. This is likely due to the complex interplay and overlap of multiple pathological processes—such as inflammation, steatosis, and fibrosis—that simultaneously influence T2 values, resulting in difficulties in distinctly separating them by T2 mapping alone. These findings are also consistent with previous clinical and preclinical studies [15, 27].

Several limitations should be acknowledged. The main limitation of this study is the absence of a liver biopsy at the time of MRI. Liver biopsy is no longer routinely conducted, and therefore was not available at the time of MRI examination. Therefore, MRE, currently considered the most accurate imaging modality for non-invasive liver fibrosis assessment, was used as the reference standard. In addition, histopathological validation of T1ρ mapping was performed in a previous preclinical study using the same imaging technique. This study focused on the clinical translation of results from this experimental study. Further, for the assessment of hepatic steatosis, PDFF was employed as a non-invasive reference standard instead of biopsy. Yet, it is also considered the most accurate non-invasive technique for quantifying hepatic fat content. Second, the selection of MRE-derived liver stiffness and PDFF to define significant fibrosis and relevant steatosis, respectively, may have influenced the results of this study. Nonetheless, the aim of this proof-of-concept study, namely to clinically translate findings from a recent animal study, was achieved. The study’s sample size, while adequate for the primary analysis, may have been underpowered for certain subgroup evaluations, which should be interpreted with caution. Future longitudinal studies with larger patient cohorts and varying cutoff values to define fibrosis and steatosis severity are necessary to investigate the diagnostic performance of T1ρ mapping and its prognostic implications. Third, the reproducibility of our findings may be limited by the specific hardware and software used in this study. Thus, further optimization and standardization of T1ρ mapping are required to facilitate broader clinical implementation of this promising technique.

In conclusion, this study shows that T1ρ mapping can serve as a more robust imaging-based biomarker for the non-invasive assessment of hepatic fibrosis across the full spectrum of CLD, including patients with SLD, compared to conventional mapping. This is also the first clinical study fully translating the findings of a recent preclinical study—validated by histopathology—into a clinical setting.

## Supplementary information

Supplementary information

## Abbreviations

AUC: Areas under the curve
CLD: Chronic liver disease
MRE: MR-elastography
PDFF: Proton density fat fraction
ROC: Receiver operating characteristic
ROI: Regions of interest
SLD: Steatotic liver disease
