# Supplementary material for: Clinical validation of T1ρ mapping for the assessment of hepatic fibrosis in patients with chronic liver disease
Source: Eur Radiol. 2025 Dec 20;36(5):3983–93. doi: 10.1007/s00330-025-12225-5 (PMC13086745; doi:10.1007/s00330-025-12225-5)
Supplement: Supplementary file 1 — Supplementary information [file 330_2025_12225_MOESM1_ESM.pdf]

Clinical validation of T1ρ mapping for the assessment of hepatic fibrosis in patients with chronic liver disease

ELECTRONIC SUPPLEMENTARY MATERIAL

Appendix S1 Inter-reader and intra-reader agreement by the assessment of MRI-derived quantitative mapping parameters in randomly chosen subset (n=50).

	**Inter-reader agreement**		
**Variable**	**ICC**	**Confidence interval**	**P value**
**Hepatic T1ρ**	0.778	0.599; 0.877	**<0.001**
**Hepatic native T1**	0.838	0.707; 0.910	**<0.001**
**Hepatic ECV**	0.805	0.647; 0.892	**<0.001**
**Hepatic T2**	0.767	0.579; 0.871	**<0.001**
	**Intra-reader agreement**		
**Hepatic T1ρ**	0.821	0.676; 0.901	**<0.001**
**Hepatic native T1**	0.865	0.756; 0.925	**<0.001**
**Hepatic ECV**	0.896	0.812; 0.942	**<0.001**
**Hepatic T2**	0.819	0.672; 0.900	**<0.001**

ICC: interclass correlation coefficient for inter-reader agreement for each quantitative MRI mapping parameter; ECV: extracellular volume fraction. Intra-reader agreement was assessed by a single reader, who repeated region of interest measurements in the same subset of 50 randomly selected MRI examinations.
